# STIGMA AND AGEISM IN THE GERIATRIC EDUCATION CONTEXT: USING PATIENT-CENTERED CARE APPROACHES

**DOI:** 10.1093/geroni/igae098.1083

**Published:** 2024-12-31

**Authors:** John Schumacher, Laura Finn

**Affiliations:** University of Maryland Baltimore County, Baltimore, Maryland, United States; Philadelphia College of Pharmacy/St. Joseph’s University, Philadelphia, Pennsylvania, United States

## Abstract

Healthcare professionals remain ill-prepared to meet the unique needs of the aging population. Yet, implementing effective geriatrics educational programming remains challenging across health care disciplines due to low participation rates and uptake. Persistent stigma, ageism, and stereotypes endemic to healthcare, and society at large, contribute to the devaluing of geriatrics content reducing the potential for effective and equitable care for older adults. Geriatric educators are challenged to address the ingrained stigma and ageism among both their learners and healthcare organizations. However, re-imagining geriatrics education to be anchored in a patient-centered care approach, addressing specific, concrete patient needs, has been shown to reduce ageist attitudes and behaviors in the healthcare workforce. Participating in intergenerational activities has also shown a positive impact on health care provider attitudes toward older patients. Furthermore, prioritizing preventive medicine and public health throughout the life course also effectively contributes to postponing the onset of disease and disability among patients. Implementing these basic educational approaches is a pathway to achieving the goal of improved outcomes for patients of all ages.

